# Supplementary material for: Chromothripsis during telomere crisis is independent of NHEJ, and consistent with a replicative origin
Source: Genome Res. 2019 May;29(5):737–49. doi: 10.1101/gr.240705.118 (PMC6499312; doi:10.1101/gr.240705.118)
Supplement: Supplemental Material [file supp_gr.240705.118_Supplemental_file_1.zip › contigs/annotated_contigs/DB53/contig.3.DB53_length_1184_mean_cov_33.4222972973.docx]

**DB53_length_1184_mean_cov_33.4222972973**

```
CCACTTCATA|CCCAACAAACCCTTTTTTTTTTTTTTTTTTTTT|TTTTT|TTTTTTTTTTTTTTTTTTTTTTTTTTTTTTTTTTTTAT
           >chr1:173963160-173963193 - E=8e-02 p=0e+00  >chr4:88485534-88486317 - E=0e
TATACTCTCAGTTTTAGGGTACATGTGCACATTGTGCAGGTTAGTTACATATGTATACATGTGCCATGCTGGTGCACTGCACCCACTAA
+00
TGTGTCATCTAGCATTAGGTATATCTCCCAATGCTATCCCTCCCCCCTCCCCCAACCCCACCACAGTCCCCAGAGTGTGATATTCCCCT

TCCTGTGCCCATGTGATCTCATTGTTCAATACCCACCTATGAGTGAGAATATGCGGTGTTTGGTTTTTTGTTCTTGCGATAGTTTACTG

AGAATGATGATTTCCAATTTCATCCATGTCCCTACAAAGGACATGAACTCATCATTTTTTATGGCTGCATAGTATTCCATGGTGTATAT

GTGCCACATTTTCTTAATCCAGTCTATCATTGTTGGACATTTGGGTTGGTTCCAAGTCTTTGCTATTGTGAATAGTGCCACAATAAACA

TACGTGTGCATGTGTCTTTATAGCAGCATGATTTATAGTCCTTTGGGTATATACCCAGTAATGGGATGGCTGGGTCAAATGGTATTTCT

AGTTCTAGATCCCTGAGGAATCGCCACATTGACTTCCACAATGGTTGAACTAGTTTACAGTCCCACCAACAGTGCAAAAGTGTTCCTAT

TTCTCCACATCCTCTCCAGCACCTGTTGTTTCCTGACTTTTTAATGATTGCCATTCTAACTGGTGTGAGATGGTATCTCATTGTGGTTT

TGATTTGCATTTCTCTGATGGCCAGTGATGA|T|TTGCATCTCCGATATCCAGAACTGTGCTTAAAAATATGTGTTTAATGAATAAATC
                                >chr17:68723043-68723398 - E=2e-183
CGTGAGTTAATTAAATGGAATCAAACAAGTACAAATACTAAATGAATATGTAAATACGAACTTTAGCTTAGTATGGTCCAATAAATTCA

ACAGACCGAAATGAAATGTGAATTCTATTTGCATGTTTTTTCCTCCTGTCCATCACATTAAAAGTATGATAGTCACAATGACAGTGATG

ATGATCATACCCAGCTCTCAAACACGCCTAATATATTTGAAGCACTAAATGCATTTTGCATATATTAACTTATTTCATATTAGATAATA

GCTACACAAATTTCAAGTTTCTTCATGGTTAA
```
